# Fast zero-shot deep learning-based denoising method for low-field MR images

**DOI:** 10.1007/s10334-025-01311-w

**Published:** 2025-12-22

**Authors:** Reina Ayde, Gabriel Zihlmann, Najat Salameh, Mathieu Sarracanie

**Affiliations:** 1 Center for Adaptable MRI Technology, School of Medicine, Medical Sciences and Nutrition, University of Aberdeen, Aberdeen, UK; 2 Department of Radiology, University of Michigan, Ann Arbor, USA

**Keywords:** MRI, Low-field, Denoising, Deep learning, Self-supervised, Zero-shot

## Abstract

**Objective:**

Denoising low-field MR images is often essential to obtain image quality that is adequate for clinical diagnosis while keeping scan time patient-friendly. The recently introduced zero-shot self-supervised approach shows great promise, requiring no prior data collection for training, which is particularly challenging at low-field. Here, this scan-specific denoising approach is adapted to low-field MR data and optimized to accelerate the training process.

**Material and method:**

We extended the zero-shot noise-as-clean method by modifying the training process to achieve faster training times. The proposed method was compared to BM4D and the recent zero-shot noise2noise methods. Denoising performance was first evaluated quantitatively on high-field data where high SNR images are available, then assessed qualitatively on prospective low-field data (0.1 T). Ultimately, we studied the denoising performance with respect to training on portions of the original data matrix as a potential strategy for further training acceleration.

**Results:**

The proposed method achieved high denoising performance across different SNR levels within a few seconds on a GPU for typical low-field data dimensions. Additionally, training on portion of the data showed potential for further training acceleration.

**Discussion:**

In the context of low-field MRI, this denoising method shows great potential, as it could be integrated into acquisition workflows relatively seamlessly to improve image quality. Code: https://github.com/reinaayde7/zs-nac.git.

**Supplementary Information:**

The online version contains supplementary material available at 10.1007/s10334-025-01311-w.

## Introduction

Low magnetic field strength MRI has experienced a renewed interest worldwide, aiming to trade complex, bulky magnets generating intense magnetic fields in clinical units for more accessible and smaller devices [1]. However, while recent results across the broad spectrum of low-magnetic field MRI look promising, the inherently impaired spin polarization limits the achievable signal-to-noise ratio (SNR) per unit time. This generally results in noisy images when targeting both clinically acceptable spatial resolutions and acquisition times, which may compromise their clinical diagnostic value.

One way to address the underlying limitations of low-field MRI is leveraging denoising approaches in a postprocessing step. The goal is to reduce image noise while both preserving the integrity of the imaged object—such as its contrast, overall shape, and details—and preventing the introduction of artifacts. In MRI, noise is well modelled by a Gaussian distribution with zero mean and variance in the real and imaginary parts of the complex image or *k*-space [2].

Over the years, various methods have been proposed for denoising MR images. These methods can be classified into analytical and learning-based approaches [3]. Analytical approaches rely on exploiting image priors such as the widely utilized non-local self-similarity prior [4, 5]. For instance, BM*x*D, considered as the gold standard traditional method of Gaussian noise, has found widespread adoption in many low-field studies not only as a denoiser but also as an evaluation standard for denoisers [6, 7].

In recent years, a paradigm shift has occurred with the emergence of learning-based techniques, also known as data-driven techniques. These emerging techniques not only demonstrate promising performance but, unlike traditional denoisers, also eliminate the need for prior assumptions. Additionally, learning-based approaches excel in terms of computational efficiency at the testing (or inference) phase, making them particularly appealing for real-time applications such as interventional MRI. Naturally, these approaches were rapidly adopted by the low-field MR community [8–12]. These studies rely on supervised learning, where the neural network learns image priors by training on an extensive dataset consisting of pairs of noisy and clean (i.e., high SNR) images. However, acquiring noisy-clean image pairs is laborious and time-consuming, particularly in low-field regimes where heterogenous magnet geometries and magnetic field strengths co-exist across different research groups. To overcome this limitation, high-field, open-source datasets were used to synthetically generate noisy and clean image pairs. However, this approach overlooks contrast differences at low-field, resulting in a domain mismatch when applied to real low-field images. Recently, Salehi et al. [13] used an open-source simulator [35] to simulate low-field brain images. While this approach can mitigate contrast issues, it is unable to account for the full diversity of MR images (anatomy, tissues, SNR, etc.). Consequently, as with all supervised learning methods, the image and noise priors learned during the training may differ from those in the testing images, leading to a domain gap or generalization problem.

Researchers have proposed unsupervised and self-supervised methods that mitigate the requirement for reference, high-SNR images [14–17]. These approaches, originally developed in computer vision, were rapidly adopted by the MR community [18, 19]. However, even without reference images, the collection of noisy images remains time-consuming and cumbersome, and training still suffers from domain gap issues.

To circumvent these limitations, zero-shot methods have been proposed that do not require a training set. Here, neural networks undergo training in a self-supervised fashion using only the designated input image for denoising. Consequently, the denoising network is tailored to a specific acquisition and overcomes challenges associated with training set collection while addressing domain gaps. Deep image prior [20], noise2void [21], self2self [22] and diffusion models such as DDM^2^ [23] are examples of zero-shot denoisers. Despite promising denoising performance, these methods can be computationally expensive (e.g. self2self, DDM^2^) or impractical to implement (e.g. deep image prior which is very sensitive to the number of iterations) [24]. Since the training phase must be executed for each image to denoise, ultra-rapid training becomes essential.

In this study, we propose a fast, scan-specific denoising method that builds upon previously communicated zero-shot self-supervised noise-as-clean (ZS-NAC) originally developed for natural images [25]. The network architecture and training were modified, and an additional input channel was incorporated for the handling of complex MRI data (i.e., real + imaginary channels). Our approach leverages a simplified U-Net architecture to accelerate training and introduces a novel strategy for terminating training upon convergence, rather than relying on an empirically determined number of epochs. Additionally, we investigated further training acceleration avenues by using only a portion of the input image and could achieved two- to tenfold acceleration with minimal impact on the quality of the denoised output.

In the absence of high-SNR (reference) in-vivo data at our low field of interest, denoising performance and training speed were first evaluated on a public high-field MR database. The denoising performance of ZS-NAC was compared to that of BM4D and the recently introduced zero-shot noise2noise (ZS-N2N) method [26]. Ultimately, ZS-NAC performance was assessed on in-vivo low-field MR data acquired at 0.1 T. Our code is publicly available as a software package and can be easily integrated into existing MRI reconstruction pipelines.

## Methods

### Theoretical background

ZS-NAC first involves adding additional noise to an already acquired, noisy image. Then, a convolutional network is trained to denoise by using the noisier image as input and the originally acquired noisy image as output (i.e., reference). Once trained, the originally acquired noisy image is fed to the trained network as input to be denoised.

In the MR context, let ${\boldsymbol{y}}={\boldsymbol{x}}+{\boldsymbol{n}}$ be the noisy acquired complex MR image, where ${\boldsymbol{x}}$ is the noise-free image and ${\boldsymbol{n}}$ is the random Gaussian noise with zero mean and ${{\boldsymbol{\sigma}}}_{{\boldsymbol{A}}}^{2}$ variance in the real and imaginary parts of the complex data. The variance ${{\boldsymbol{\sigma}}}_{{\boldsymbol{A}}}^{2}$ is estimated by computing the noise standard deviation of the real and imaginary parts over the entire background of the MR complex image, from a computed binary mask excluding the imaged object (cf. Methods Experiment 1). Then, multiple noisier MR images ${\boldsymbol{z}}={\boldsymbol{y}}+{\boldsymbol{m}}$ are created by adding noise ${\boldsymbol{m}}$ drawn from a noise distribution with zero mean and ${{\boldsymbol{\sigma}}}_{{\boldsymbol{B}}}^{2}$ variance with ${{\boldsymbol{\sigma}}}_{{\boldsymbol{B}}}=\boldsymbol{\alpha }{{\boldsymbol{\sigma}}}_{{\boldsymbol{A}}}$**,** where α is a scalar number (set to 1 in ZS-NAC). Subsequently, a deep learning network ${\boldsymbol{f}}$ is trained to predict ${\boldsymbol{y}}$ given ${\boldsymbol{z}}$ as an input. Once trained, the estimated clean image ${\boldsymbol{x}}\boldsymbol{^{\prime}}$ is predicted using ${{\boldsymbol{x}}}^{\boldsymbol{^{\prime}}}={\boldsymbol{f}}({\boldsymbol{y}})$**.**

Another popular method, noisier2noise [16], employs a similar strategy with a difference occurring in the inference phase. However, in a zero-shot context, we found that the ZS-NAC method led to better stability and image sharpness, particularly at low SNR levels (see supplementary materials A). Therefore, we proceeded with the ZS-NAC method.

### Training details

**Fig. 1:**
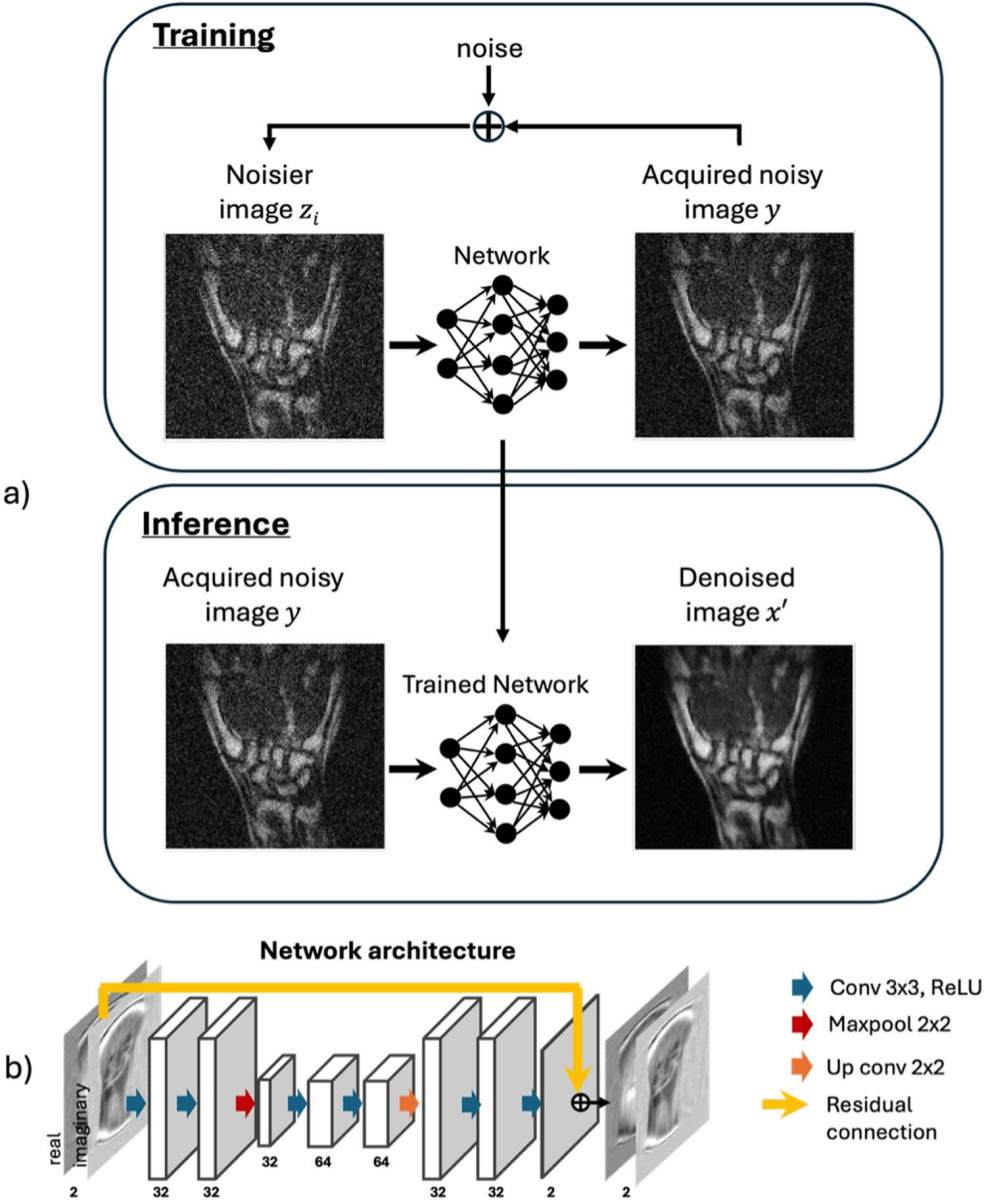
**a** Schematic explaining the ZS-NAC approach used here. The first step consists of training the convolutional network to predict y given z_i_. The second step, or inference step, consists of using the trained network to denoise y. **b** Network structure: The two input/output channels correspond to the real and imaginary parts of the image. The network consists of a single downsampling/upsampling step. The downsampling step consists of two convolution operations with 32 features (size: 3 × 3, stride: 1) followed by a Maxpooling operation (size: 2 × 2, stride: 2). Then, two convolution operations with 64 features are executed followed by an upsampling operation. This last step is achieved by a transpose convolution followed by two additional convolutional operations. All convolution operations are followed by a rectified unit (ReLU) and Batch normalization operation

The absence of training set is balanced by the generation of multiple pairs (${{\boldsymbol{z}}}_{{\boldsymbol{i}}}$**,** ${\boldsymbol{y}}$) where ${{\boldsymbol{z}}}_{{\boldsymbol{i}}}$ is a noisier image simulated out of the noisy image ${\boldsymbol{y}}$ at each epoch ${\boldsymbol{i}}$. Subsequently, a convolutional neural network is trained to denoise the inputs ${{\boldsymbol{z}}}_{{\boldsymbol{i}}}$. Once trained, the network is used to denoise the input ${\boldsymbol{y}}$ (Fig. 1a).

In the proposed work, ZS-NAC was modified to achieve a fast-denoising process. The choice of the network architecture is crucial for achieving both fast and accurate denoising. A fast training requires a simple network architecture, so a modified light version of residual U-Net network was adopted with a total of 101 k trainable parameters (Fig. 1b). The network consists of a single downsampling/upsampling step. The downsampling step consists of two convolution operations with 32 features (size: 3 × 3, stride: 1) followed by a Maxpooling operation (size: 2 × 2, stride: 2). Then, two convolution operations with 64 features are executed followed by an upsampling operation. This last step is achieved by a transpose convolution followed by two additional convolutional operations. All convolution operations are followed by a rectified unit (ReLU) and Batch normalization operations.

One challenge of zero-shot learning is the absence of validation set, which not only ensures an unbiased evaluation of the model but also helps drastically reducing the training time by stopping it once convergence is achieved. In reference [25], training was stopped after an arbitrary number of epochs. In this work, a validation pair was generated by downsampling the noisy image using the kernel = $$\left[\begin{array}{cc}0& 1\\ 0& 0\end{array}\right]$$, and creating a noisier version of this small image by adding noise drawn from a noise distribution with zero mean and ${{\boldsymbol{\sigma}}}_{{\boldsymbol{B}}}^{2}$ variance. This allowed to adopt an early stopping criterion essential to speed the overall process. One might assume that a simple noisier image could serve as a validation set. However, this would result in a loss equivalent to the training loss.

The complex nature of MR images was preserved by splitting real and imaginary data into 2 channels. Additionally, an ablation study of the effect of loss function used during the training was done (see supplementary material C). As a result, Mean Squared Error (MSE) was used as a loss function to train the network. Adam optimizer with an initial learning rate value of 0.01 was adopted. The learning rate gradually decreased by a factor of 0.9 when the training loss did not improve. A batch number equal to the number of slices in a 3D acquisition was adopted for training. A software package was developed for installation using PyPI, ensuring easy deployment. The code and installing instructions are available at https://github.com/reinaayde7/zs-nac.git.

### BM4D in MRI

The ZS-NAC method was compared to BM4D. BM4D is an extension of BM3D for volumetric data. It operates by grouping similar patterns/patches present in the image into a 3D array and then filtering the latter using sparse representations in the transform domain [4, 27]. As BM*x*D is an analytical method, it does not require a database.

For pure Gaussian noise, BM*x*D is considered as one of the gold-standard methods [3, 24]. It is however important to avoid applying conventional BM*x*d to magnitude MR images, especially for low-field data, where noise distribution is more likely to follow a Rician distribution. BM*x*D can also handle Rician distributions using a variance-stabilizing transformation as previously communicated [4, 28]. In this work, we used the conventional BM4D python package for volumetric, multichannel data applied to our complex MR data.

The standard deviation of the underlying noise in the real and imaginary channels was estimated per data sample and used as an input parameter for BM4D. This standard deviation determines the threshold value when filtering coefficients in the transform domain. In practice, using the standard deviation led to non-optimal smoothed images. Therefore, we added a factor θ<1, that multiplies the input standard deviation for optimal performance. To identify the θ leading to the best denoising performance, we compared quantitatively the performance for different SNR levels and identified the best θ value (see supplementary materials B). Accordingly, θ = 0.9 was adopted in all experiments.

### Zero-shot noise2noise

We further compared our method to the latest, zero-shot denoising technique, known as zero-shot noise2noise (ZS-N2N), which offers relatively fast denoising times [26]. According to the authors, denoising a 256 × 256 image took 20 s on a GPU Quadro RTX 6000. Two downsampled images are generated from the acquired noisy image using two fixed filters. A small convolutional network (21 k parameters) is then trained to map one downsampled image to the other. This method builds upon noise2noise [15] and neighbor2neighbor [14] approaches, minimizing computational resources while preserving image quality. We adapted this network to handle two input channels (real and imaginary). Mansour et al. argue that there is no need for an early stopping criterion because a consistency loss was used which prevents overfitting. The hyperparameters used in their shared notebook was used for training. The maximum number of epochs was set to 2000 as the convergence is reached between 1000 and 2000 epochs.

## Experiments

### Performance assessment on fastMRI data

Since reference or high-SNR data are hard to obtain at low-field, MR images acquired at 3-T from the fastMRI dataset [29] were used to quantitatively evaluate the method performance. Nine sets of brain MR images obtained from different sequences (i.e., with different contrasts) were used in total: three sets correspond to FLAIR acquisitions, three to T1-weighted (T1w) acquisitions and three to T2-weighted (T2w) acquisitions. All multicoil 2D MR data were converted into single coil data using coil sensitivity estimation ESPIRiT [30] followed by coils combination via least square solution [31]. Original matrix sizes of the data were: T1w [640 × 320 × 14], T2w [768 × 396 × 16], and FLAIR [640 × 320 × 16]. The oversampling by a factor of 2 in the readout direction was removed. Additionally, to enhance further the SNR of those images, the *k-*space was cropped in-plane to increase the voxel size and thus the SNR. The matrix size of each image set was set to [256 × 128 × 14].

Subsequently, Gaussian noise was added to the real and imaginary parts of these images to generate noisy images ${\boldsymbol{y}}$ with a specific SNR level ranging between 10 and 40. In this work, we define the SNR in magnitude image as the ratio of the average highest 1%-pixel intensities to the standard deviation of the noise.

The performance of the proposed approach was compared to BM4D and ZS-N2N. Since deep learning approaches are non-deterministic, slight variations in the denoised image are expected. To evaluate whether it remains acceptable within a clinical workflow, ZS-NAC and ZS-N2N denoising approaches were repeated 10 times to assess repeatability.

Denoising performance was assessed using structure similarity index (SSIM) (Wang et al., 2004) and peak SNR (PSNR). A binary mask defined as a region-of-interest (ROI) was generated for each image. First, a signal intensity thresholding was applied to the normalized magnitude image. Then, a sequence of morphological image processing techniques (erosion and dilation) was applied to generate a background mask for each slice. This mask was used to exclude background contributions when computing SSIM and PSNR, as including them might skew the evaluation of denoising performance within the ROI. Additionally, this mask was used to estimate the noise variance ${{\boldsymbol{\sigma}}}_{{\boldsymbol{A}}}^{2}$.

### Performance assessment on low-field MRI data

The performance of this approach was evaluated qualitatively on prospective 3D *in-vivo* human wrist data acquired with a 0.1 T unshielded, non-commercial extremity scanner [32, 33]. Data were collected using a spoiled gradient-echo-based sequence with the following parameters: matrix size = [128 × 128 × 15], voxel size = [1 × 1 × 2.9] mm^3^, and echo time TE = 6 ms. Two repetition times TR = 13 ms and 55 ms along with corresponding numbers of averages NEX = 3 and 10, were used to produce varying image contrasts. The resulting image SNR ranged from 15 to 25. The performance of the proposed approach was compared to BM4D and ZS-N2N.

### Total processing time

The full duration of processing was monitored, including training and inference, on the nine brain fastMRI sets with a fixed matrix dimension of [256 × 128 × 14] and SNR ranging from 10 to 40. It was then compared to BM4D and ZS-N2N. As BM4D is not available on GPU, the processing time of the different denoising approaches was assessed on both CPU and GPU. Testing was performed on an RTX 4090 Nvidia GeForce GPU (24 GB of RAM) and an Intel Core i9-13900 K 3 GHz CPU (128 GB of RAM). The experiment was repeated 10 times for ZS-NAC and ZS-N2N and the average denoising speed (in kilopixels/second or kp/s) across sets and repetitions was reported.

### Matrix size performance dependency

In a single coil setting, which is common at very low field (≤ 0.1 T), noise is spatially invariant through the image set. This feature can be used to accelerate further the training process, notably when dealing with large data matrices. In this experiment the training step was performed only on a portion of the input matrix while inference (i.e., denoising) was still performed on the full data matrix. We assessed the effectiveness of this approach by studying the dependence of matrix portion size (i.e., input number of pixels) on denoising performance.

First, we conducted experiments on T1-, T2-weighted and Flair MR data with a full matrix size of [320 × 320 × 16], [384 × 396 × 16] and [320 × 320 × 16], respectively. The size in the third dimension was reduced by symmetrically cropping from the center, reducing the size by 10% to 90% in 10% increments. Accordingly, the resulting numbers of slices were rounded, such that instead of using 16 slices, the network was trained on 1, 3, 5, …, up to 15 slices. In-plane cropping is equally relevant. However, careful selection of the patch region is required, ensuring it includes a no-signal region for accurate noise standard deviation estimation. Inference (i.e., denoising) was performed on the full matrix size image. Batch size was set to the number of slices the network was trained on. Each training was repeated 10 times. SSIM and training duration were reported as a function of the number of pixels the network was trained on.

Then, a similar experiment was carried out on *in-vivo* brain dataset acquired on a non-commercial 0.1 T whole-body system modified from a MAG4.3 scanner (Sopha-Magnetech, Paris, France). A 3D gradient-spoiled GRE sequence was used with the following parameters: TE/TR: 6/11 ms, resolution = [2 × 2 × 2] mm^3^, number of averages = 1. The acquired matrix size was [128 × 128 × 136]. The size of the third dimension was varied by symmetrically cropping from the center, with reductions ranging from 10 to 90% in 10% increments. Batch size was set to 16. Qualitative assessment of ZS-NAC network was evaluated for different input matrix sizes.

## Results

### Performance assessment on fastMRI data

**Fig. 2:**
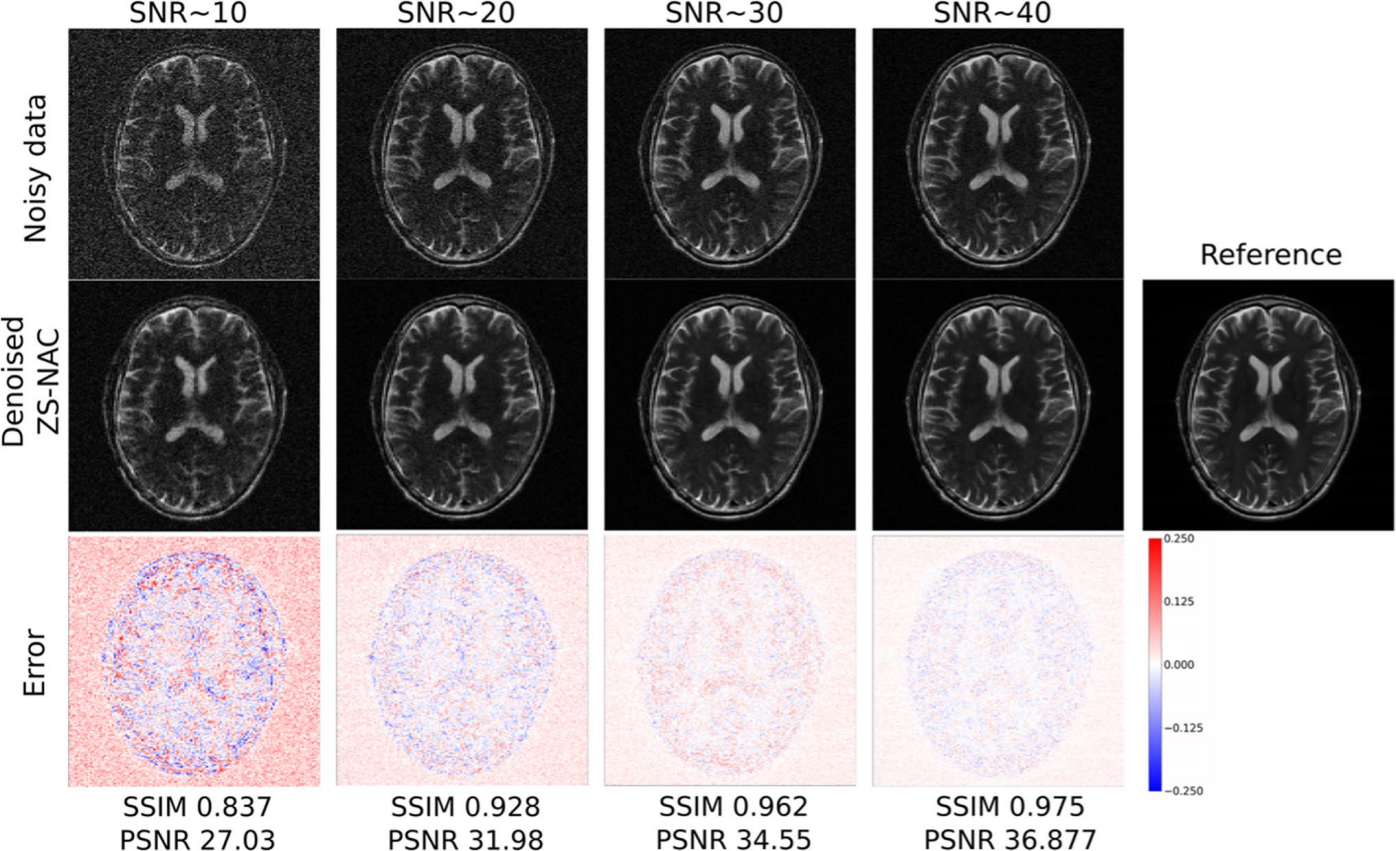
An example of ZS-NAC performance on a T2-weighted fastMRI brain image for different simulated SNR levels: 10, 20, 30 and 40. The upper row shows the noisy input magnitude images. The middle row displays the ZS-NAC denoised images. The bottom row presents the difference between the denoised and the reference magnitude images. All images are normalized to [0, 1] scale

**Fig. 3:**
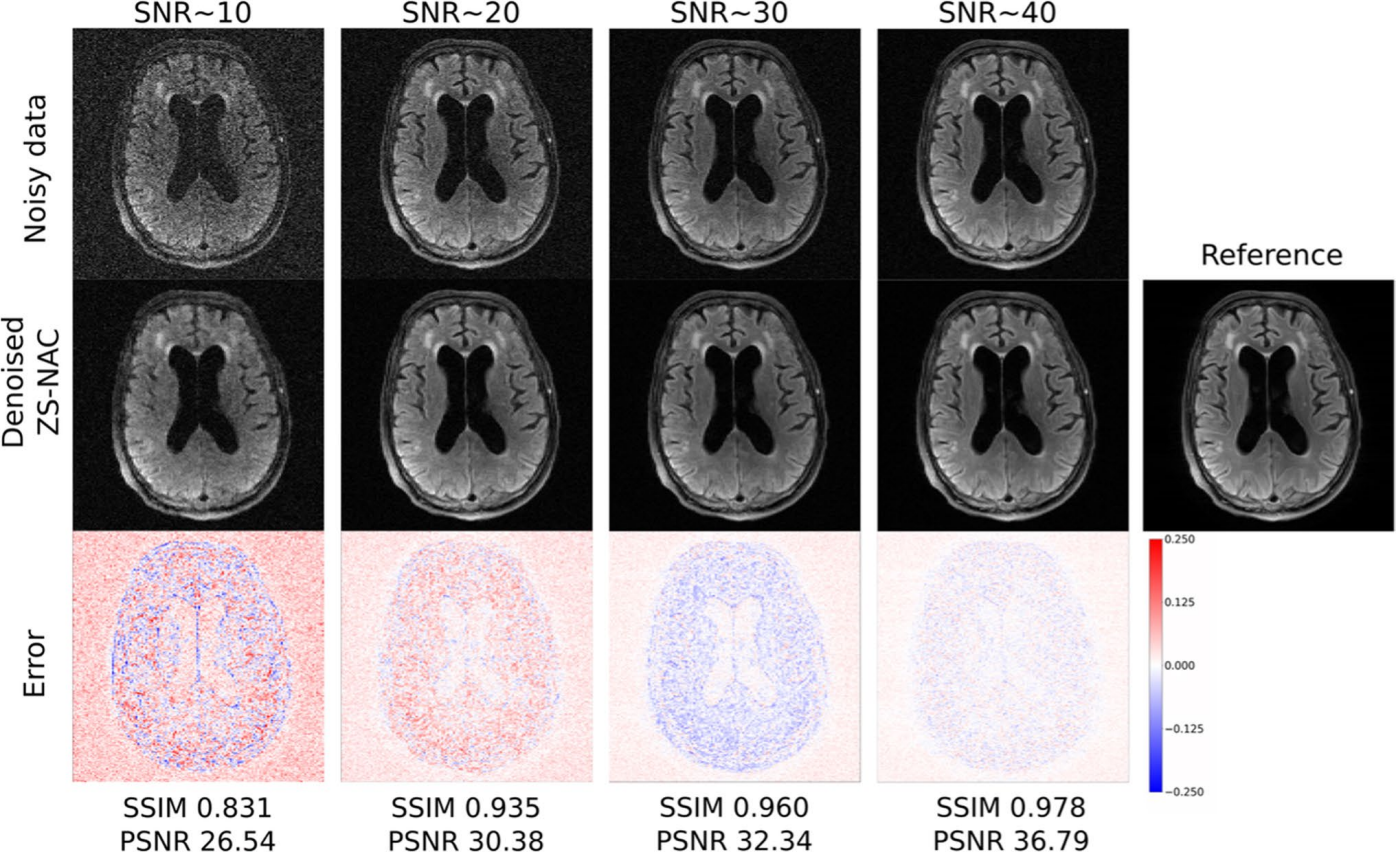
An example of ZS-NAC performance on a FLAIR fastMRI brain image for different simulated SNR levels: 10, 20, 30 and 40. The first row shows the noisy input magnitude images. The second row displays the ZS-NAC denoised images. The bottom row presents the difference between the denoised and the reference magnitude images. All images are normalized to [0, 1] scale

Figures 2 and 3 illustrate denoising performance on the brain fastMRI data with various SNR levels (10 to 40) and for two different contrasts, respectively. Qualitatively, ZS-NAC effectively denoises images while preserving the overall texture, contrast and details for SNR > 20. However, as SNR decreases, performance diminishes, and noise remains within the ROI.

**Fig. 4:**
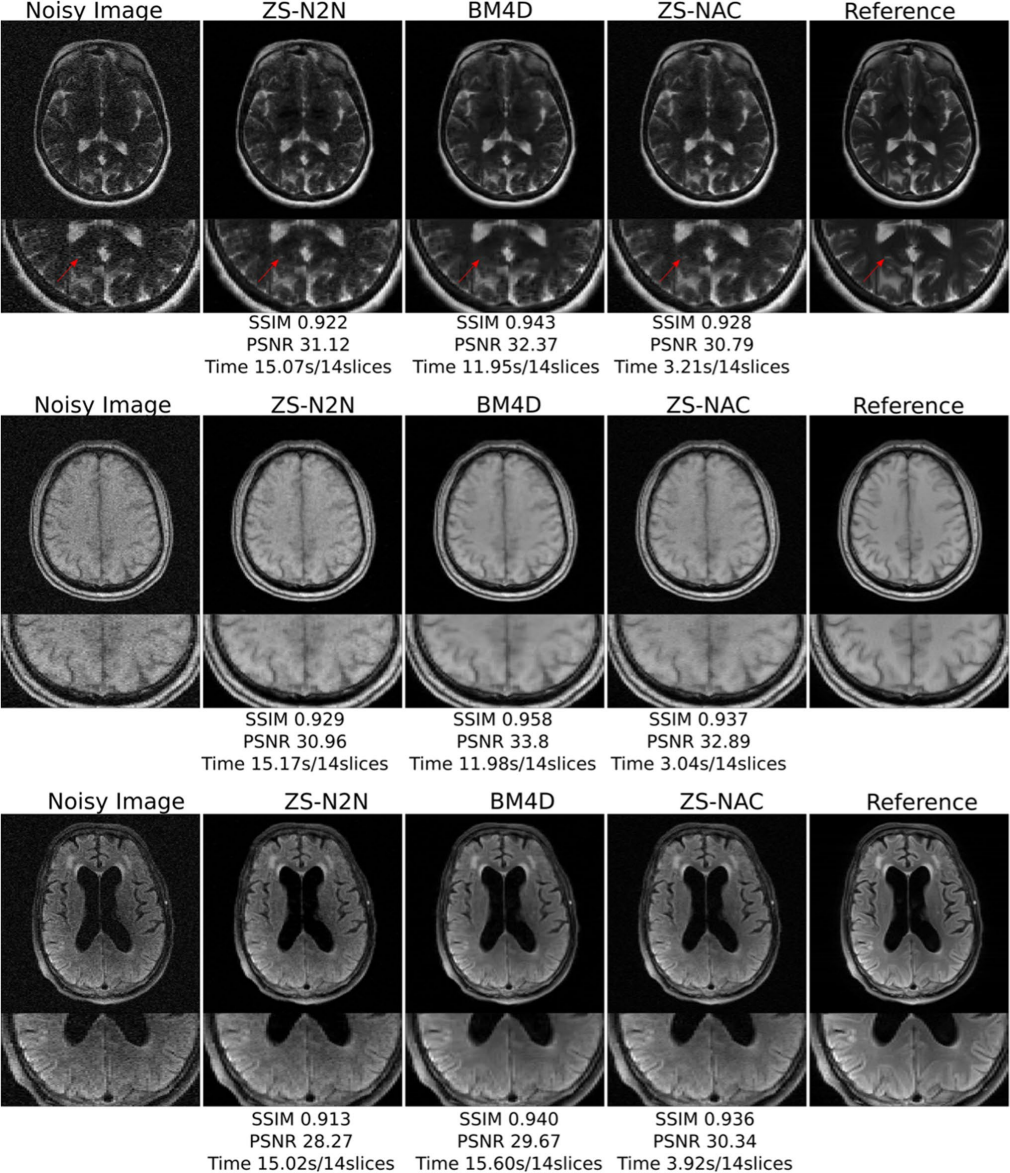
Comparing ZS-NAC denoising performance with ZS-N2N and BM4D on simulated, noisy fastMRI brain data. SNR of the noisy images is approximately 20, and image contrasts are respectively T2-, T1-weighted and FLAIR (top, middle, and bottom rows). The red arrows in the first row point to a small structure smoothed out by BM4D. All images are normalized to [0, 1] scale

**Fig. 5:**
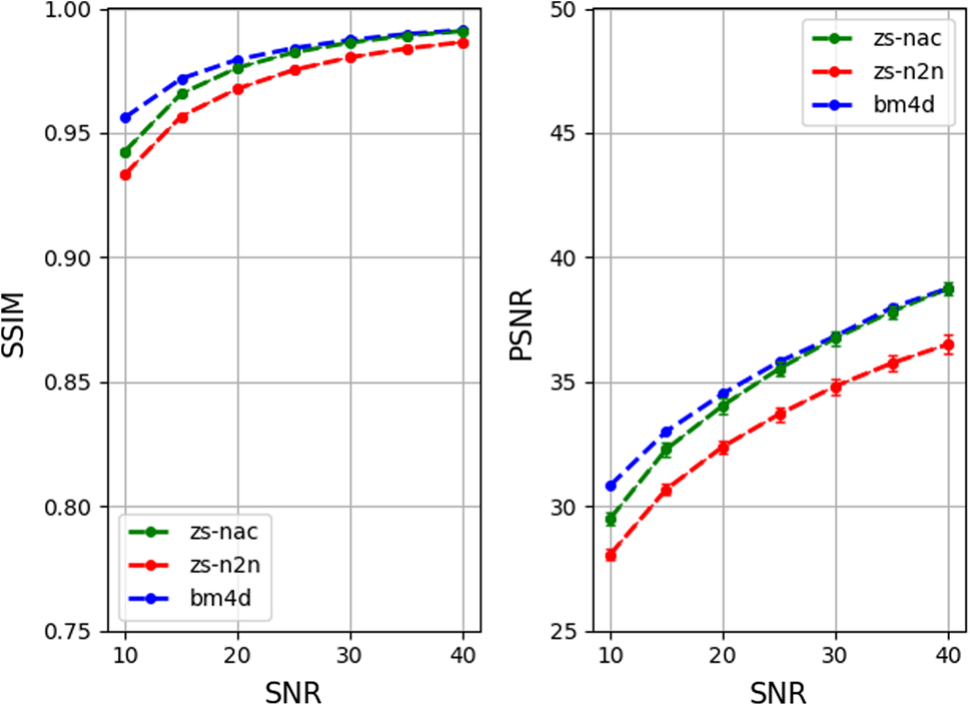
Mean (± standard deviation) SSIM and PSNR scores of ZS-NAC, ZS-N2N and BM4D denoising methods obtained on 9 sets of fastMRI data at various simulated SNR levels. The experiments were repeated 10 times for both ZS-NAC and ZS-N2N2 to assess the methods repeatability

Figure 4 provides examples of the proposed approach’s performance compared to ZS-N2N and BM4D on the brain fastMRI data with different image contrasts and SNR ~ 20. Qualitatively, ZS-N2N demonstrates high capability at preserving details, though at the expense of leaving residual noise in the ROI. BM4D demonstrates strong denoising performance but often results in oversmoothing, as seen in the region indicated by the red arrow, where a fine detail has been lost (red arrow in Fig. 4). ZS-NAC shows a good balance between details preservation and denoising. Quantitatively, Fig. 5 shows that BM4D has the best metrics for SNR levels ≤ 25. For SNR > 25, ZS-NAC and BM4D achieve comparable metrics while ZS-N2N has lower metrics across all SNR levels. The mean standard deviation across the 9 sets for a given SNR in Fig. 5 indicates the repeatability of ZS-NAC and ZS-N2N denoising processes. The standard deviation of SSIM is negligible for both ZS-N2N and ZS-NAC. Additionally, the mean standard deviation for PSNR is consistently much lower than 1% for both ZS-N2N and ZS-NAC across all SNR levels.

### Performance assessment on low-field MRI data

**Fig. 6:**
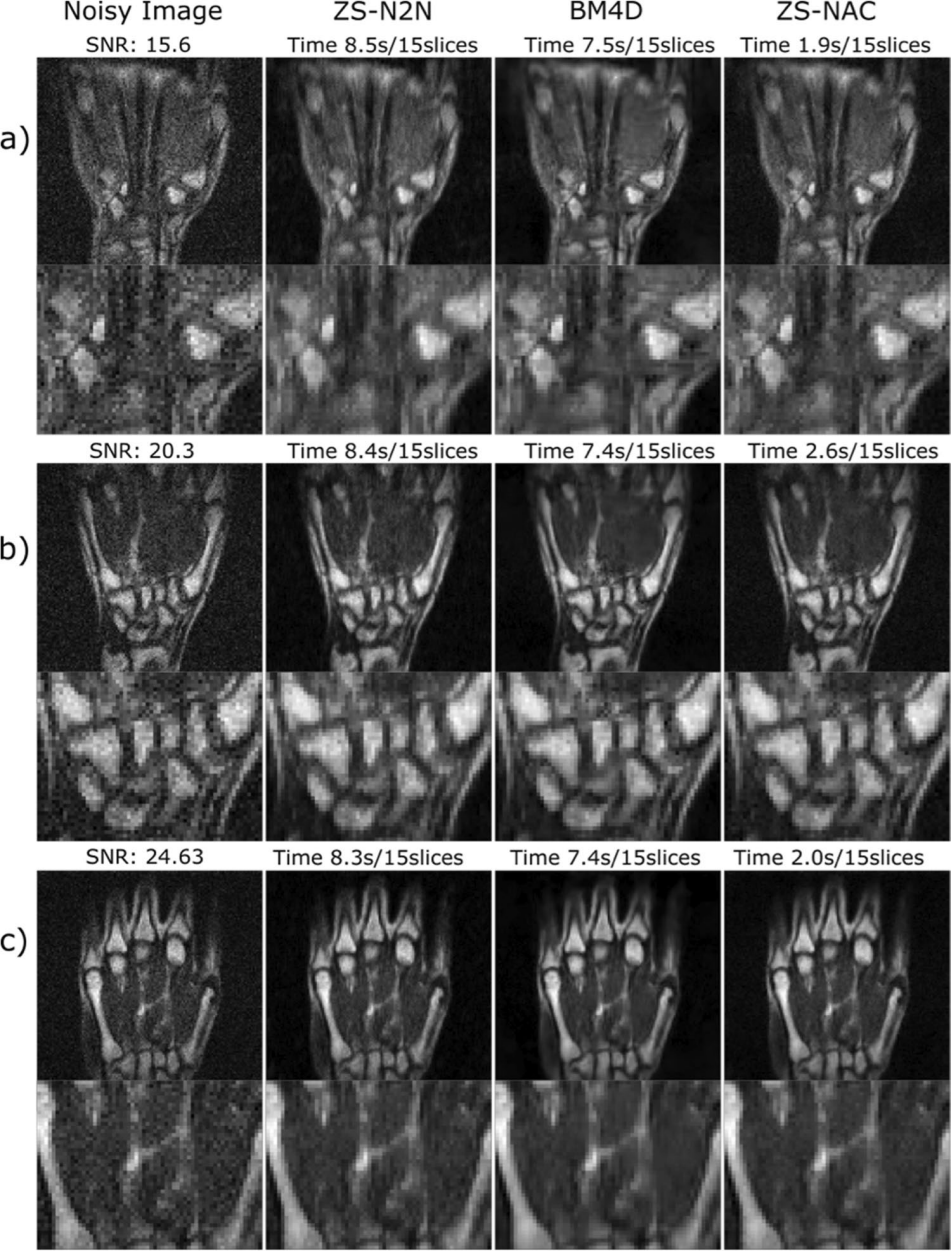
Comparison of ZS-NAC, ZS-N2N with BM4D for different wrist data acquired on a low-field, unshielded 0.1 T scanner. **a** Matrix size = [128 × 128 × 15], TE/TR = 7/55 ms, voxel size = [1 × 1 × 2.9] mm^3^, and Nex = 3. **b** Matrix size = [128 × 128 × 15], TE/TR = 7/13.8 ms, voxel size = [1 × 1 × 2.9] mm^3^, and Nex = 10. **c** Matrix size = [128 × 128 × 15], TE/TR = 7/13.8 ms, voxel size = [1 × 1 × 2.9] mm^3^, and Nex = 10. All images are normalized to [0, 1] scale

Figure 6 compares the different denoising approaches on prospectively acquired hand/wrist low-field data with a matrix size of [128 × 120 × 15] and different SNR levels ranging from 15 to 25. We observe trends comparable to the high-field data. BM4D produces rather smoothed images, while ZS-N2N provides sharper images but requires a relatively long processing time compared to ZS-NAC. The latter offers good balance between denoising quality and computation time.

### Total processing time

**Fig. 7:**
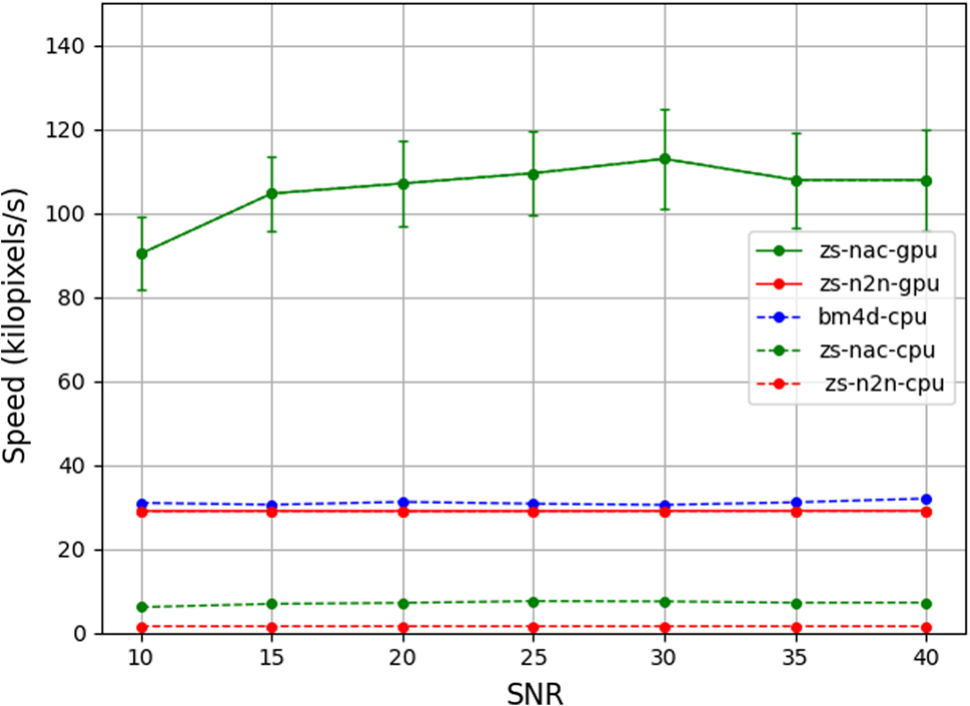
Average denoising speed (kilopixels/s) for different denoising methods on CPU and GPU for a matrix size of [256 × 128 × 14] with varying SNR levels ranging from 10 to 40

We monitored the computational times for denoising fastMRI data with a fixed matrix size of [256 × 128 × 14] and SNR ranging from 10 to 40. The average denoising speed (kilopixels/s) across the sets and repetitions is reported in Fig. 7. On a GPU, ZS-NAC achieves the highest speed, being ~ 3.6 × faster than ZS-N2N (run on GPU) and ~ 3.4 × faster than BM4D (run on CPU) for SNR ≥ 15. For SNR ≤ 15, ZS-NAC is slightly slower due to more epochs needed to converge. The observed variance in speed for the ZS-NAC approach ~± 10% is attributed to the non-deterministic nature of the process resulting in a varying number of epochs needed for convergence. In contrast, since there is no early stopping in ZS-N2N, the duration/speed does not vary between repetitions. The duration of ZS-N2N training process is given by the epoch duration and the maximum number of epochs. Epoch duration depends on network size as well as the input matrix size. For a fixed matrix size of [256 × 128 × 14], the time per epoch for ZS-N2N is 7 ms, while the time per epoch for ZS-NAC is 20 ms. It is important to note that there is a GPU launch overhead time that takes ~ 1 s depending on the set up (hardware and software). The reported training speed excludes this time by adding a ‘warm up’ step to the GPU before starting the actual training.

On a CPU, BM4D is unsurprisingly faster than ZS-N2N and ZS-NAC for matrix size of [256 × 128 × 14], since CPUs are not effective at handling simultaneous computational tasks necessary for training deep neural networks.

Table 1 presents the denoising duration (seconds) and speed (kilopixels/s) of the different denoising methods at a fixed SNR of 20 and different matrix sizes. As expected, larger matrices require more time for denoising across all methods. For typical low-field data size of [128 × 128 × 14], ZS-NAC takes only ~ 2 s on a GPU, and about ~ 22 s on a CPU. For a single slice image, ZS-NAC denoising is achieved within hundreds of milliseconds on a GPU and ~ 2.5 s on a CPU, which is comparable to BM4D’s processing duration. Speed wise, GPUs generally achieve higher speed (or better time efficiency) when processing larger-scale matrices as they have non-negligeable kernel launch overhead. For matrices exceeding 100 kilopixels, ZS-NAC achieves a processing speed of ~ 100 kilopixels/s, compared to ~ 30 kilopixels/s for a 16 kilopixels matrix.

**Table 1:** Time required for denoising a set of T2-weighted brain fastMRI data with a SNR level set to 20 and varying matrix sizes. The speed is computed by dividing the number of pixels by the denoising time

	[Matrix size] # pixels (kp)	[128 × 128 × 1] 16 kp	[256 × 256 × 1] 65 kp	[128 × 128 × 14] 229 kp	[256 × 128 × 14] 458 kp	[256 × 256 × 14] 971 kp
GPU	ZS-NAC	0.54±0.1 s	0.76±0.11 s	1.94±0.16 s	3.96±0.25 s	10.05±1.53 s
		29.91±5.68 kp/s	86.12±13.53 kp/s	118.13±9.92 kp/s	115.78±7.53 kp/s	91.29±14.30 kp/s
	ZS-N2N	4.39±0.05 s	4.70±0.02 s	8.41±0.08 s	15.48±0.05 s	29.45±0.07 s
		3.72±0.04 kp/s	13.91±0.07 kp/s	27.26±0.26 kp/s	29.62±0.11 kp/s	31.15±0.08 kp/s
CPU	ZS-NAC	2.41±0.18 s	6.54±0.42 s	21.25±1.83 s	61.48±4.79 s	188.47±15.28 s
		6.79±0.51 kp/s	10.01±0.65 kp/s	10.78±0.93 kp/s	7.46±0.58 kp/s	4.86±0.39 kp/s
	ZS-N2N	38.00±0 s	59.17±0 s	143.57±0 s	288.20±0 s	593.25±0 s
		0.43±0 kp/s	1.10±0 kp/s	1.59±0 kp/s	1.59±0 kp/s	1.54±0 kp/s
	BM4D	1.96±0 s	2.42±0 s	7.88±0 s	13.95± 0. s	25.40± 0 s
		8.35±0 kp/s	26.99±0 kp/s	29.08± 0 kp/s	32.88± 0. kp/s	36.11± 0 kp/s

### Matrix size performance dependency

**Fig. 8:**
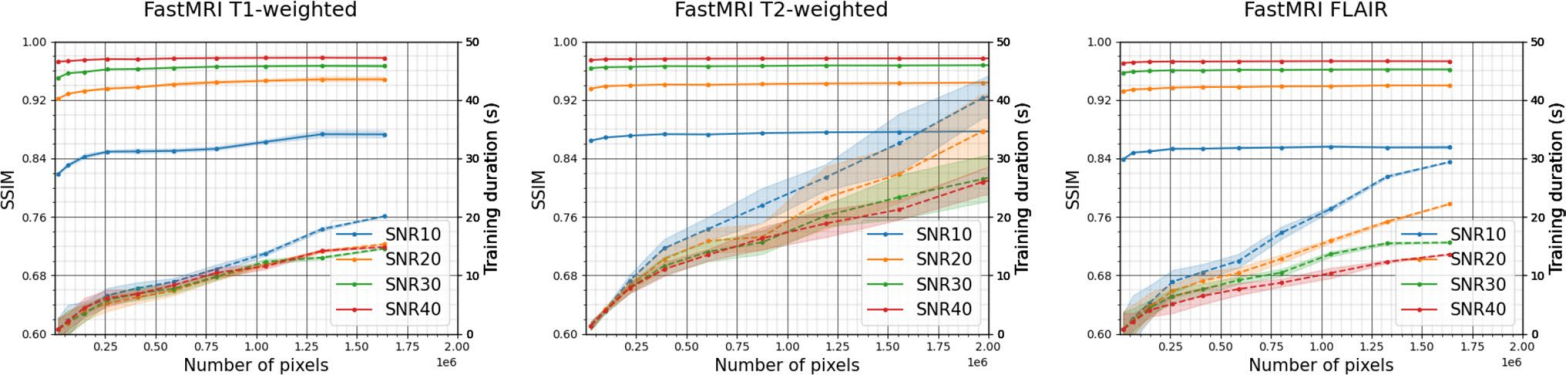
SSIM values (solid lines) and training duration (dashed lines) of ZS-NAC denoised images as a function of the number of training pixels (or matrix size). The results were obtained on 3 sets of FastMRI data: T1-weighted, T2-weighted, and FLAIR at various simulated input SNR levels: 10, 20, 30 and 40

**Fig. 9:**
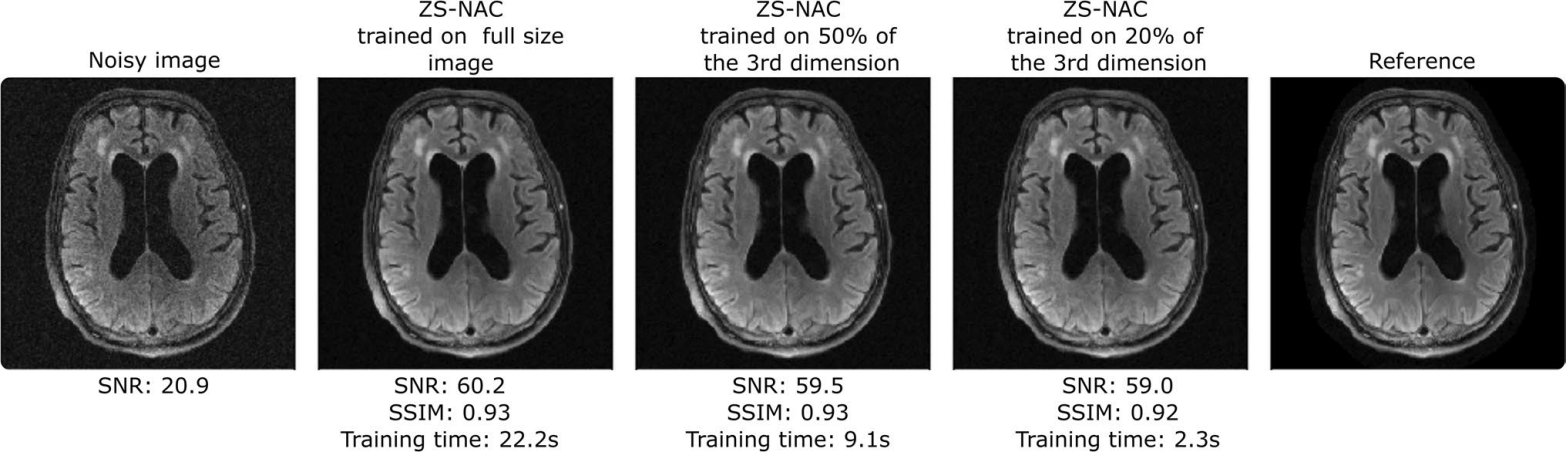
Examples of ZS-NAC denoising of fastMRI FLAIR data with an original matrix size = [320 × 320 × 16] using, respectively, 100%, 50% and 20% of the slices for training. All images are normalized to [0, 1] scale

**Fig. 10:**
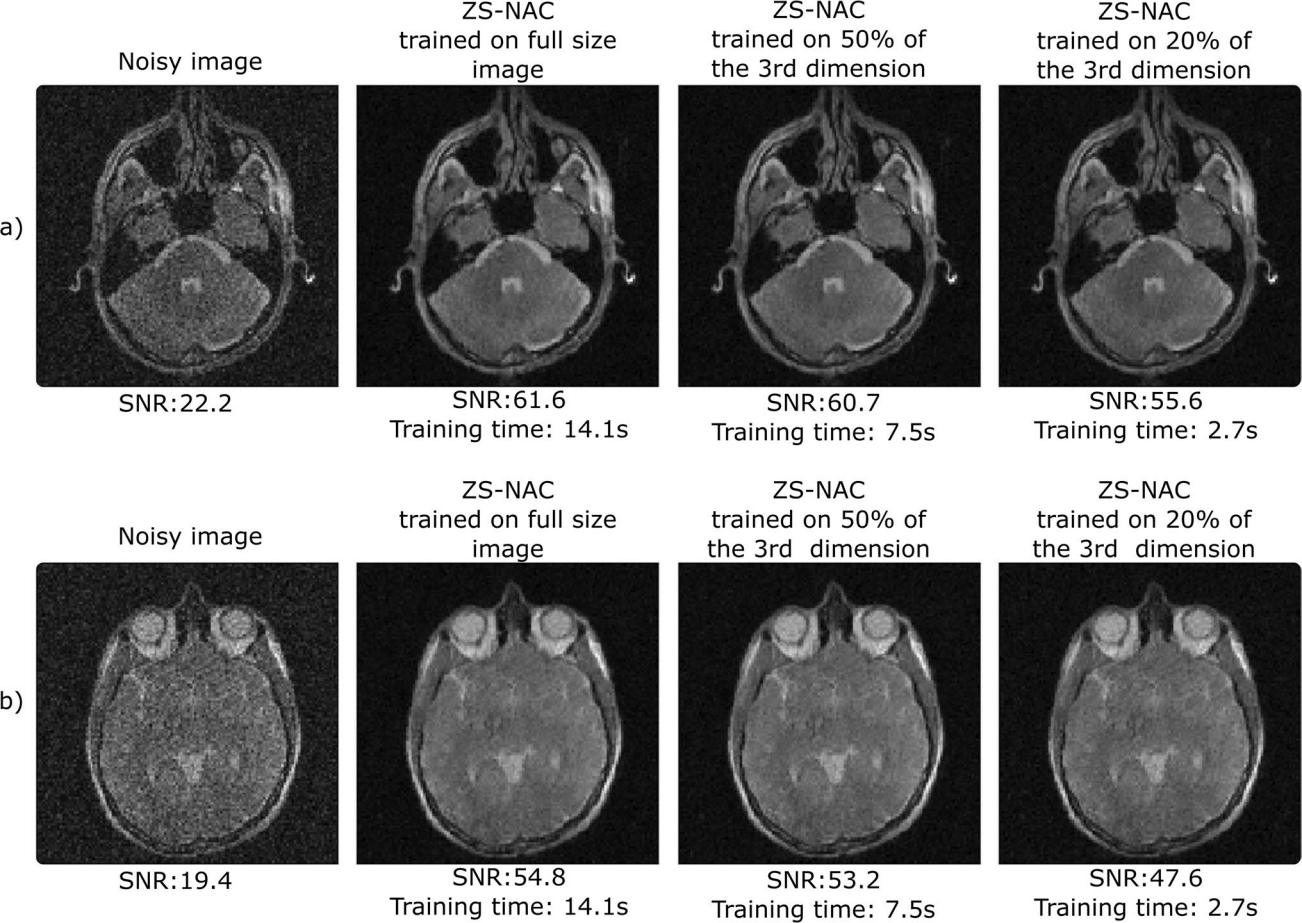
Examples of ZS-NAC denoising of a low-field MR data with matrix size = [128 × 128 × 136] using for training a 100%, 50% and 20% of the slices for training. All images are normalized to [0, 1] scale

Figure 8 shows the mean SSIM (± standard deviation) of the denoised images and the mean training duration (± standard deviation) as a function of the number of input pixels (or matrix portion size) used for ZS-NAC training, across different brain MR image contrasts and input SNR levels. As shown, increasing the number of input pixels (i.e., using a larger training matrix) improves SSIM across all data and input SNR levels. However, the improvement is subtle and follows an asymptotic trend beyond a certain threshold —except for SNR = 10 in T1-weigted images. In contrast, training time increases almost linearly with the number of input pixels, indicating that a significant reduction in training time—by at least a factor of two—can be achieved with minimal impact on SSIM. Figure 9 presents an example from the fastMRI FLAIR dataset, demonstrating the denoising performance of ZS-NAC when trained on reduced input matrix size. Training on only 20% of the third dimension (through-plane direction) leads to an SSIM drop of approximately 1%, while achieving a 10 × acceleration in training time. Figure 10 shows a similar example on 0.1 T, low-field brain data. Although SSIM is not reported due to the absence of reference images, the plateauing effect is reflected in the SNR of the denoised outputs. Notably, training on only 50% of the third-dimension results in an SNR decrease of just ~ 1.5%, while achieving a reduction in training time of approximately ~ 47%. Even with just 20% of the third dimension, the results remain qualitatively acceptable, achieving a ~ 5 × acceleration.

## Discussion

In the proposed work, we adapted and refined noise-as-clean zero-shot denoising (ZS-NAC) to achieve fast denoising while maintaining satisfactory performance on high- and low-field complex MR data. The approach does not require a database, overcoming typical challenges of data-based learning such as extra data collection and domain gap issues. The small convolutional network together with early stopping allowed training to be achieved within a few seconds.

Network performance was first assessed on fastMRI data, where high-SNR data could be generated and multiple SNR levels simulated. Despite reasonable overall results, some residual noise can remain in the ROI, particularly for low SNR input images (SNR < 20). Nevertheless, since no blurring or artifacts are introduced, the ZS-NAC approach remains a compelling choice. For SNR ≥ 20, ZS-NAC demonstrates rather satisfactory denoising performance competing with BM4D.

Quantitatively, BM4D appears to denoise images more effectively at SNR < 20 but do tend to smooth edges and present patchy areas. In contrast, ZS-N2N preserves edges and details best but leaves some noise in the ROI. ZS-NAC presented here appears to offer a good balance between the two approaches. Interestingly, quantitative metrics indicate a preference toward smoother data, as BM4D achieves the highest scores, whereas ZS-N2N shows lower scores due to higher residual noise in the ROI. This highlights the importance of interpreting quantitative metrics with caution, as it may not fully correlate with radiologists perception who often prefer noisier images with preserved details over an overly smoothed image introducing a risk of losing information [19, 34]. A detailed radiological evaluation was not performed in this work and will be an important aspect to consider in future studies. Another benefit of our method is its consistent behavior—as the standard deviation of the quantitative metrics across 10 repetitions is insignificant. This consistency combined with its ability to alleviate the burden of data collection and generalization issues, makes ZS-NAC a trustworthy choice for medical applications.

It is worth noting that the choice of the θ factor in BM4D was based on quantitative metrics. As mentioned, these metrics might be favorable to smoother images. Therefore, further fine-tuning of θ based on perceptual appreciation might be needed to improve BM4D performances.

Similarly, ZS-NAC performance may be further optimized by fine-tuning α in ${{\boldsymbol{\sigma}}}_{{\boldsymbol{B}}}=\boldsymbol{\alpha }{{\boldsymbol{\sigma}}}_{{\boldsymbol{A}}}$ that was set to 1 throughout this work. For instance, a higher α will produce a higher level of denoising but with the risk of blurring details.

Unlike ZS-N2N, ZS-NAC and BM4D are not ‘blind’ denoising methods. This means that the noise distribution should be well-known for an optimal performance. The proposed work is limited to Gaussian noise distributions, which is acceptable for MRI provided that the input images are complex (i.e., real and imaginary data), cartesian, and fully sampled (without *k-*space zero-filling). The non-linear magnitude transformation modifies the distribution of the noise from Gaussian to Rician particularly for low-SNR data [2]. Therefore, ZS-NAC is less suitable for magnitude MR images unless SNR levels are high enough for noise to be approximated as Gaussian. However, this method could certainly be adapted by modifying the added noise distribution—e.g., replacing Gaussian with Rician noise—to make it suitable for the processing of magnitude low-field data.

Speed wise, when a GPU is available, ZS-NAC demonstrates the fastest processing time, reaching few hundreds of milliseconds per slice for typical low-field data dimensions. Such capability is appealing as it allows denoising to be integrated into image processing pipelines, without being a bottleneck in future potential routine clinical pathways.

Finally, we investigated the impact of training on a portion of image matrix as a potential way to further accelerate the training step. Our results indicate that the number of pixels ZS-NAC is trained on, which is closely related to the training time, and the achieved SSIM or SNR improvement, do not scale proportionally. While training times increase steadily with the number of pixels, gains in image quality plateau beyond a certain point. This allows a substantial reduction in training time —by more than a factor of two and up to a factor of ten—while incurring only a modest decrease in SSIM or SNR. This approach could also potentially be advantageous in real-time applications such as interventional low-field MRI.

## Conclusion

In conclusion, we adapted and improved ZS-NAC to achieve reliable MRI denoising performance within short computational times. Unlike most supervised and self-supervised approaches, this scan-specific approach does not require a training database and is not affected by domain gap issues. We validated this method and compared it to both BM4D and ZS-N2N using simulated high-field MR data as well as prospectively acquired 0.1 T low-field data with varying SNR levels. Our approach significantly reduced noise while preserving image details and without introducing artifacts. The small convolutional network coupled with early stopping resulted in a relatively short processing times: a few seconds on a GPU and ~ 1 min on a CPU for a matrix size of [256 × 128 × 14]. In the context of low-field MRI, this denoising method shows great potential, as it could be integrated into acquisition workflows relatively seamlessly to improve image quality.

## Supplementary Information

Below is the link to the electronic supplementary material.

Supplementary file1 (DOCX 2967 KB)

## Data Availability

The code is shared on GitHub. The data that support the findings of this study are available on request from the corresponding author (R. Ayde) for non-commercial, research purposes.
