# Supplementary material for: Fast zero-shot deep learning-based denoising method for low-field MR images
Source: MAGMA. 2025 Dec 22;39(4):531–44. doi: 10.1007/s10334-025-01311-w (PMC13447554; doi:10.1007/s10334-025-01311-w)
Supplement: Supplementary file 1 [file 10334_2025_1311_MOESM1_ESM.docx]

Supplementary materials

A. Zero-shot noisier2noise VS noise-as-clean

The main difference between the standard noisier2noise and noise-as-clean (ZS-NAC) methods lies in the inference stage. In noisier2noise, during inference, additional noise $\boldsymbol{m}$ sampled from the same noise distribution as the acquired image $\boldsymbol{y}$ is added producing an image twice as noisy $\boldsymbol{z=y+}\boldsymbol{m}$. The latter is fed to the trained deep neural network $\boldsymbol{f}\left( \boldsymbol{.} \right)$. A correction step is then applied to remove the remaining noise from the output image. The final denoised image $\boldsymbol{x}^{\boldsymbol{'}}$ is given by:

$$\boldsymbol{x}^{\boldsymbol{'}}\boldsymbol{=}\boldsymbol{2}\boldsymbol{f}\left( \boldsymbol{z} \right)\boldsymbol{-}\boldsymbol{z}$$

Since further degrading of the acquired image might not desirable during inference, Moran *et al.* proposed an improvement to the standard method. They suggested adding noise with a variance $\boldsymbol{\sigma}_{\boldsymbol{B}}\boldsymbol{=\alpha}\boldsymbol{\sigma}_{\boldsymbol{A}}$ where $\boldsymbol{\sigma}_{\boldsymbol{A}}$ is the noise variance of the acquired image and $\boldsymbol{\alpha}$ is a factor smaller than 1. This reduces the noise added to the acquired image compared to the case where noise is doubled. Inference is then performed using the following equation:

$$\boldsymbol{x}^{\boldsymbol{'}}\boldsymbol{=}\frac{\boldsymbol{[}\left( \boldsymbol{1}\boldsymbol{+}\boldsymbol{\alpha}^{\boldsymbol{2}} \right)\boldsymbol{f}\left( \boldsymbol{z} \right)\boldsymbol{]-}\boldsymbol{z}}{\boldsymbol{\alpha}^{\boldsymbol{2}}}$$

In this work, we compared the improved version of noisier2noise with $\boldsymbol{\alpha=0.5}$ (as suggested in Moran *et al.*) to ZS-NAC method on three high-field datasets with different contrasts (T1w, T2w and FLAIR). The same experiment was repeated 10 times to assess the repeatability.

Denoising performance and reproducibility were assessed both qualitatively and quantitatively (PSNR and SSIM). It should be noted that noisier2noise was not originally developed in a zero-shot approach. Therefore, we modified noiser2noise training details to match those of ZS-NAC.

Figure S1: Comparison of denoising performance and repeatability of ZS-NAC (top block) and zero-shot (ZS)-noisier2noise (bottom block) approaches. Black arrows indicate instability that might occur with ZS-noisier2noise method. All images are normalized to [0, 1] scale.

Figure S2: Comparing accuracy performance of zero-shot (ZS)-noisier2noise and zs-noise-as-clean (ZS-NAC). Denoising was done on three different sets of high-field data (T1-, T2-weighted and FLAIR images) and repeated 10 times.

Figure S1 demonstrates that both methods show good denoising capabilities. However, noisier2noise shows instability in performance when the same process is repeated (black arrows in figure S1) and often generates smoother images.

Figure S2 shows that SSIM results for ZS-noisier2noise and ZS-NAC are comparable, but ZS-NAC achieves a higher PSNR than ZS-noisier2noise. Additionally, the standard deviation of the latter is higher, indicating that ZS-NAC is more stable than ZS-noisier2noise.

B. Setting BM4D standard deviation factor $\theta$

Figure S3: Search for the noise standard deviation factor $\theta$ maximizing the quantitative metrics (PSNR and SSIM) of BM4D. Three experiments were conducted for different high-field data contrasts (T1-, T2-weighted and FLAIR) and different SNR levels (10, 20, 30, 40).

The BM4D function uses the input noise standard deviation ($\sigma$) to determine the threshold for filtering coefficients in the transform domain to attenuate noise. This significantly impacts the quality of the denoised image and can be fine-tuned to balance denoising and detail preservation. Fine-tuning can be done by multiplying the standard deviation by a factor $\theta$. To determine the best $\theta$, quantitative metrics (PSNR and SSIM) were recorded for three different high-field brain datasets with varying contrasts (T1w, T2w and FLAIR) and SNR levels (10, 20, 30 and 40). The results are shown in figure S3. While small changes can be observed with varying SNR and image contrast, the best value according to the graphs is $\theta$ = 0.9.

C. Ablation study on loss function

An ablation study on loss function was conducted. Denoising performance of ZS-NAC was compared on fastMRI T2-weighted brain data (simulated SNR=20) using three different training losses: MSE, MAE, and SSIM. ZS-NAC Denoising was repeated ten times for each loss function. An example of the results is shown in figure S4, and table S1 summarizes the quantitative results averaged over the ten repetitions. According to table S1, MSE and MAE loss functions achieved comparable performance in terms of SSIM, PSNR, and training time whereas the SSIM loss function resulted in lower quantitative metrics and longer training time. The longer training time is expected, as SSIM involves more computational steps than MSE and MAE, and its relatively lower performance has been discussed in Zhao et al.(Zhao et al. 2017). Based on these findings, we selected the MSE loss function for subsequent experiments.

Figure S4: An example of ZS-NAC performance using different loss functions: SSIM, MAE and MSE performed on a T2-weighted FastMRI brain image with simulated SNR levels of 20.

|  | MSE loss | MAE loss | SSIM loss |
| --- | --- | --- | --- |
| SSIM | 0.950$\pm$0.0008 | 0.950$\pm$0.0007 | 0.932$\pm$0.0026 |
| PSNR | 31.550$\pm$0.220 | 31.450$\pm$0.224 | 29.607$\pm$0.311 |
| Speed (kp/s) | 107.72$\pm7$.96 | 114.520$\pm$4.88 | 53.165$\pm$13.296 |

Table S1: Quantitative results and training speeds of ZS-NAC denoising using different loss functions. The mean and standard deviation were computed on T2-weighted FastMRI data with a simulated SNR level of 20 over 10 repetitions.

**References:**

Zhao, Hang, Orazio Gallo, Iuri Frosio, and Jan Kautz. 2017. “Loss Functions for Image Restoration With Neural Networks.” *IEEE Transactions on Computational Imaging* 3 (1): 47–57. https://doi.org/10.1109/TCI.2016.2644865.
